# 3D Printed Graphene Based Energy Storage Devices

**DOI:** 10.1038/srep42233

**Published:** 2017-03-03

**Authors:** Christopher W. Foster, Michael P. Down, Yan Zhang, Xiaobo Ji, Samuel J. Rowley-Neale, Graham C. Smith, Peter J. Kelly, Craig E. Banks

**Affiliations:** 1 Faculty of Science and Engineering, Manchester Metropolitan University, Chester Street, Manchester M15 GD, UK; 2 College of Chemistry and Chemical Engineering, Central South University, Changsha 410083, China; 3 Faculty of Science and Engineering, Department of Natural Sciences, University of Chester, Thornton Science Park, Pool Lane, Ince, Chester CH2 4NU, UK

## Abstract

3D printing technology provides a unique platform for rapid prototyping of numerous applications due to its ability to produce low cost 3D printed platforms. Herein, a graphene-based polylactic acid filament (graphene/PLA) has been 3D printed to fabricate a range of 3D disc electrode (3DE) configurations using a conventional RepRap fused deposition moulding (FDM) 3D printer, which requires no further modification/*ex-situ* curing step. To provide proof-of-concept, these 3D printed electrode architectures are characterised both electrochemically and physicochemically and are advantageously applied as *freestanding* anodes within Li-ion batteries and as solid-state supercapacitors. These *freestanding* anodes neglect the requirement for a current collector, thus offering a simplistic and cheaper alternative to traditional Li-ion based setups. Additionally, the ability of these devices’ to electrochemically produce hydrogen via the hydrogen evolution reaction (HER) as an alternative to currently utilised platinum based electrodes (with in electrolysers) is also performed. The 3DE demonstrates an unexpectedly high catalytic activity towards the HER (−0.46 V *vs.* SCE) upon the 1000th cycle, such potential is the closest observed to the desired value of platinum at (−0.25 V *vs.* SCE). We subsequently suggest that 3D printing of graphene-based conductive filaments allows for the simple fabrication of energy storage devices with bespoke and conceptual designs to be realised.

Over the recent decade there has been an acceleration of interest in the fabrication and application of advanced 2D nanomaterials, such as; graphene^1^,^2^, quantum dots^3^,^4^, molybdenum disulphide^5^ and boron nitride^6^. Research into 2D nanomaterials has been driven by their enhanced physical properties over that of their macroscopic counterparts. These beneficial physical properties have permitted the utilisation of 2D materials to be regularly applied within an array of energy generation/storage devices.

Currently, there has been a natural progression towards the design and fabrication of complex structures *via* the utilisation of 3D printing. 3D printing has the ability to provide a beneficial platform for the creation of low cost 3D components for an array of applications^7^. Electrochemical 3D systems have recently been explored, however there has been a particular focus upon the utilisation of metallic printed structures for applications such as supercapacitors^8^ and microfluidic devices^9^. In respect to 3D printed battery storage, the first micron 3D printed Li-ion battery was introduced by Sun *et al*.^10^ utilising lithium-based composites Li_4_Ti_5_O_12_ (LTO) and LiFePO_4_ (LFP), using a direct-ink writing protocol with corresponding specific capacity values of 131 and 160 mAh g^−1^ respectively. Fu *et al*.^11^ have also considered this approach with the ‘3D printing’ of the a full Li-ion cell, with a graphene oxide ink bound to LTO and LFP as the cathode and anode material respectively, exhibiting similar specific capacities as Sun *et al*.^10^. The 3D printing of a *fully* graphitic-based ‘ink’ has also been recently considered by Zhu *et al*.^12^ whom comprise a 3D printable aerogel *via* a direct-ink writing protocol containing graphene oxide and graphene nanoplatelets for application as a supercapacitor. This 3D printed aerogel is reported to exhibit a capacitance of 4.79 F g^−1^ at a current density of 0.4 A g^−1^ within an aqueous solution of 3 M KOH, deduced utilising the weight of the full device.

These direct-writing protocols are useful, however in the majority of scenarios the *in-situ* curing and layering of the ‘ink’ is far from ideal for the creation of *freestanding* 3D printed electrochemical systems^13^. For example García-Tuñon *et al*.^14^ incorporate the freezing of their sample with liquid nitrogen after extrusion/printing and prior to application. True 3D printing technology, as presented here, allows for the creation of a structure that can be utilised without any further complicated post-curing/fabrication processes. Therefore, the fabrication and partial characterisation of graphite-based polylactic acid (PLA) and acrylonitrile-butadiene-styrene (ABS) conductive filaments (with graphene loadings of up to 5.6% *wt*.) have been reported by Wei *et al*.^15^ whom successfully print through a relatively low cost fused deposition modelling (FDM) 3D printer. In addition, Symes *et al*.^16^ have created the first fully 3D printed electrochemical cell using a low cost 3D printer, in which carbon black working and counter macroelectrodes have been 3D printed for electrosynthetic applications. Not only is this fabrication methodology being used for laboratory reaction vessels, Rymansaib *et al*.^17^ have utilised a 3D printed electrode as a potential electrochemical sensor for the detection of lead (II) within an acidic aqueous solution. Such elegant work has identified a potential scope for the creation of low cost and advantageous electrochemical platforms *via* a conventional 3D printing fabrication method.

This paper reports, for the first time, the utilisation of 3D printable electrochemical energy storage architectures using a graphene-based PLA filament (graphene/PLA) fabricated/printed using a conventional RepRap FDM 3D printer (shown in Fig. 1A–C) explored as a potential graphene-based lithium-ion anode and solid-state graphene supercapacitors. Furthermore, the ability to electrochemically produce hydrogen, *via* the hydrogen evolution reaction (HER), as an alternative to commonly utilised platinum based electrodes currently utilised within electrolysers is demonstrated.

## Physicochemical Characterisation of the Graphene/PLA Filament and the Printed Three-Dimensional Electrodes (3DE)

In order to benchmark this new electrochemical platform, the physicochemical properties of the graphene/PLA and the printed 3DE are first considered *via* an array of characterisation techniques.

First, the thermal properties of the graphene/PLA filament are compared with an industry standard PLA *via* thermogravimetric analysis (TGA). ESI Fig. 1 depicts a phase transition of the industry standard PLA, graphene/PLA and the 3D printed 3DE over the temperature range of 25–800 °C, where it is clear that the graphene/PLA starts to thermally degrade at a much lower temperature than that of the industry standard PLA, 160 °C and 300 °C respectively. Additionally, upon reaching the maximum temperature the residual weight percentage of the graphene/PLA corresponds to ~10%, compared to that of the industry standard of less than 1%. The printed 3DE exhibits similar thermoplastic characteristics as its graphene/PLA form, however the residual weight has decreased to a value of ~8%. These findings suggest that the fabrication and the resulting printing of this filament will have an negligible effect upon its overall thermal properties and the percentage of active material within the printed structure.

**Figure 1:**
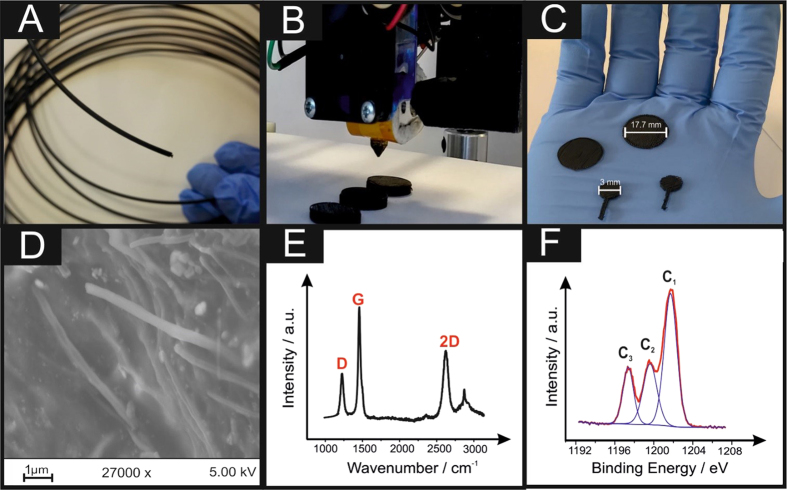
Optical images of the 3D printable graphene/PLA (**A**), the 3D printing process (**B**) and a variety of printed 3DEs used throughout this study (**C**). Corresponding SEM (**D**), Raman (**E**) and XPS analysis of the printed 3DE are also presented.

Next, the surface uniformities of the graphene/PLA filament and the 3DE were examined utilising scanning electron microscopy (SEM). ESI Fig. 2 presents SEM images of a cross section of the graphene/PLA filament where it is clear that the surface is not uniform as there are large areas of crystalline material embedded within the surface. ESI Fig. 2C,D demonstrate that there is an array of PLA nanowires present upon the surface of the filament, which has not been 3D printed. Surface analysis of the printed 3DE is next considered. ESI Fig. 3A,B indicate that upon printing of this filament into a useful structure, the surface appears to possess less uniformity of the graphene/PLA, with cracks and ridges being created. Further magnification (Fig. 1D and ESI Fig. 3C,D) of these areas depict similar nanowires (as seen previously), however it is clear that the PLA structure/binder is more prevalent than the previous SEM images. Further surface analysis was conducted utilising energy-dispersive X-ray spectroscopy (EDS) within ESI Fig. 4, it is clear that carbon and oxygen are the most predominant peaks, typically from a combination of the PLA structure and the graphene-like nature of this structure. Intriguingly, for all the samples examined (graphene/PLA filament and printed 3DE) the presence of titanium is apparent.

Raman analysis was performed (Fig. 1E) upon the printed 3DE, the signals are not typical of pristine or even *quasi*-layer graphene, with characteristic graphitic D, G and 2D peaks at 1250, 1500 and 2700 cm^−1^ respectively. Additional comparative analysis of the industry standard PLA and the graphene/PLA have also been undertaken and are presented within ESI Fig. 5A, where it is clear that the graphene/PLA and 3DE are comparable. It is postulated that the graphene in the PLA filament mixture is agglomerated in the form of multi-layer graphene. This is confirmed with full width half maximum (FWHM) analysis of the 2D peak where values of 81 and 94 cm^−1^ are determined for graphene/PLA and the printed 3DE respectively, which are much higher values than that of monolayer or *quasi*-layer graphene, where values correspond to 28 cm^−1 ^ 18 and 58 cm^−1 ^ 19 respectively. To further analyse the presence of the titanium depicted within the EDS, ESI Fig. 5B presents a Raman spectra over an earlier region of wavenumbers, *i.e.* 100–1000 cm^−1^, it is evident that there is an inclusion of peaks that are characteristic of titania (TiO_2_) at 145, 190, 400, 650 cm^−1^ similar to TiO_2_ samples recently analysed by Leong *et al*.^20^. From inspection of this Raman analysis it is difficult to decipher if the TiO_2_ belongs to either an anatase or brookite arrangement (probably mixed phase). It is important to note that the additional peak present at 890 cm^−1^ is due to the amorphous silica (*i.e.* glass slide) used for analysis.

X-ray photospectroscopy (XPS) analysis of the printed 3DE was next compared in terms of its atomic carbon and oxygen content, depicted in Fig. 1F is the spectrum for carbon 1 s. The peaks denoted C_1_, C_2_ and C_3_ are typical of the chemical composition of PLA, however it has been reported by Vergne *et al*.^21^ that PLA characteristically possesses comparative peak areas for each of these peaks. Therefore, the amplified C_1_ peak indicates an increase within the presence of non-oxygenated carbon bonds (*i.e.* C-C), presumably from the incorporation of graphene into the structure. Evaluation of the deconvoluted XPS analysis is represented within Table 1 for the 3DE, which is benchmarked against an industry standard PLA filament and the graphene/PLA filament. Overall, it is clear that the 3D printing of this graphene/PLA results in an increase of oxygenated species upon its surface, most probably due to the change of temperature within the 3D printing process.

## Electrochemical Characterisation of the 3D Graphene/PLA Filament and 3D printed 3DEs

**Figure 2:**
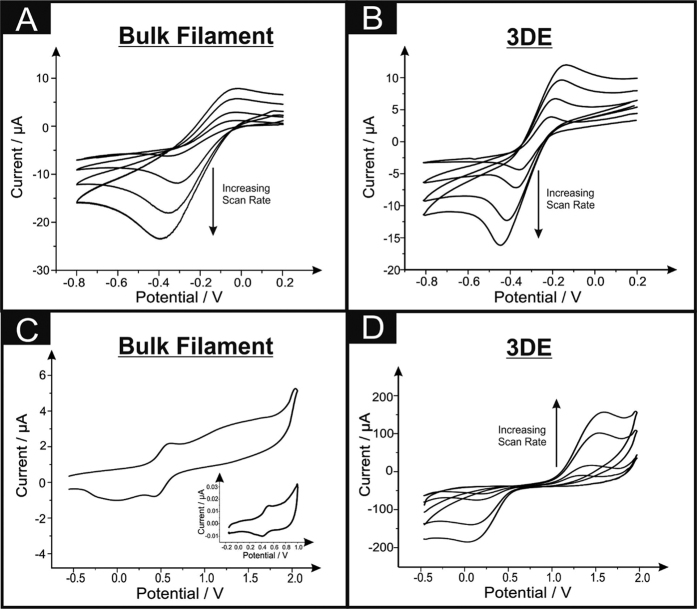
Cyclic voltammetric (*vs*. SCE) analyses over a range of scan rates (5–200 mV s^−1^) of the graphene/PLA filament and printed 3DE within a 1 mM hexaammineruthenium (III) chloride/0.1 M KCl (**A** and **B** respectively) and 1 mM ammonium iron (II) sulfate/0.2 M HClO_4_ (**C** and **D** respectively). Inset of C is the cyclic voltammetric response from a blank 0.2 M HClO_4_ solution utilising the graphene/PLA filament.

Electrochemical characterisation of the fabricated 3DEs and graphene/PLA filament using the redox probe hexaammineruthenium (III) chloride was next undertaken and benchmarked against literature. The utilisation of this probe has been chosen due to its outer-sphere redox mechanism that is insensitive to the C/O ratio groups and is affected only by the electronic structure of the 3DE (*i.e.* edge plane like-sites/defects)^22^,^23^ and is a commonly utilised redox probe in the academic literature. Voltammetric analysis over a range of scan rates were next studied utilising the 3DE and graphene/PLA filament towards 1 mM hexammineruthenium (III) chloride/0.1 M KCl and are depicted in Fig. 2A,B respectively. Interestingly, when the filament is in its bulk form the voltammetric responses exhibit sigmoidal behaviour (especially at lower scan rates), however upon printing of a 3DE the voltammetry demonstrates a *quasi*-reversible system over the chosen scan rates (5–500 mV s^−1^), as the peak-to-peak separation is over that of 59 mV. Further analysis of this data was carried out in the form of a plot of log_10_ *I*_*p*_ *vs.* log_10_ *ν* for the graphene/PLA and printed 3DE, exhibiting gradients of 0.44 and 0.42 respectively, where such values are expected for the case of a semi-infinite diffusion model, with no presence from thin-layer effects^24^. The heterogeneous rate transfer constants, *k*^*0*^_*obs*_, were deduced using both electrode platforms (as described in the Methods section). The *k*^*0*^_*obs*_ values for were found to correspond to 1.00 × 10^−3^ cm s^−1^ and 4.58 × 10^−4^ cm s^−1^ for the graphene/PLA filament and the 3DE indicating a smaller amount of edge plane sites when compared with that of other traditional graphitic-based electrodes, with the graphene/PLA exhibiting faster electron kinetics than that of the 3DE. The observed *k*^*0*^_*obs*_ for graphitic-based electrodes has regularly been shown within the literature to be contributed from two planes of the graphitic material; *firstly* the edge plane sites and *secondly* the basal plane sites. However, it is well reported that the edge plane sites are vastly superior in terms of electron transfer (*ca.* 0.4 cm s^−1^) than their basal plane counterparts (*ca.* 10^−9^ cm s^−1^). Therefore, it is commonly understood that the *k*^*0*^_*obs*_* = k*^*0*^_*edge*_(*θ*_*edge*_), where *θ*_*edge,*_is the amount of edge sites present on the electrode surface as reported by Hallam *et al*. and Davies *et al*.^24^,^25^. Taking these factors into consideration the amount of edge active sites is estimated for the graphene/PLA and the printed 3DE, with edge plane percentage values corresponding to 0.25% and 0.11% respectively, exhibiting a relatively low amount of edge sites when compared to pristine graphene platelets described by Hallam *et al*.^24^ whom report an edge plane percentage value of 2.55%. Nevertheless, it is important to note that the graphene/PLA system presented here only possesses a maximum of ~8% of graphene (confirmed *via* TGA previously). Next, Table 2 reports the variation in the electrochemical area of the graphene/PLA and 3DE, from the Randles-Ševčík equation described within the Methods Section and compares the calculated geometrical surface area. It is quite clear that the graphene/PLA possesses more electrochemically effective areas than that of the printed 3DE, this is postulated to be due to the graphene within a filament being tightly packed over a small geometric area, upon printing the same amount/percentage of graphene is spread over a larger area.

In order to understand the surface orientated groups residing upon the graphene/PLA and the 3DE, analysis using redox probe ammonium iron (II) sulfate in 0.2 M HClO_4_ (Fe^2+^_(*aq*)_) was also considered^23^,^26^. This inner-sphere probe is well known to be extremely sensitive to the carbonyl groups upon the electrode’s surface and thus can help determine the electrochemical surface characteristics of these electrode platforms. Figure 2C,D depict cyclic voltammetric responses over a range of scan rates utilising both the graphene/PLA filament and the printed 3DE. Intriguingly, when the graphene/PLA filament is solely used towards Fe^2+^_(*aq*)_, there is no substantial voltammetric signal present. However, it is clear that a redox couple is present between ~+0.3 to +0.5 V (*vs.* SCE), which is also existent within the blank solution of HClO_4_ (see inset of Fig. 2C). It is inferred that this contamination could be from the fabricated graphene used within the manufacture of this conductive filament and is unlikely to come from other origins. Upon investigation of Fig. 2D it is apparent that when the filament is 3D printed there is a large increase within the voltammetric current, supporting our prior hypothesis confirmed *via* XPS analysis, that there are increased amounts of oxygenated surface groups present upon the surface of the printed 3DE.

The sole analysis of the printed 3DE was next considered using a selection of analytes that are affected from a mixture of electronic properties and the surface orientated groups upon the electrodes. First, we analyse NADH within a pH 7.4 PBS. Banks and Compton^27^ have reported that NADH is predominantly affected by electronic properties only. Utilisation of an edge plane pyrolytic graphite electrode, which possess a high proportion of edge plane sites, allows the electrochemical oxidation peak potential to occur at ~+0.5 V (*vs*. SCE)^27^. The cyclic voltammetric oxidation of NADH is depicted in ESI Fig. 6A, where a large voltammetric signature is present at ~+0.8 V (*vs*. SCE), however upon repetition of this procedure the 3DE demonstrates signs of severe adsorption/fouling of the electrode surface. Clearly, there is an increase in the peak potential from ~+0.5 V (*vs*. SCE) (utilising traditional graphitic-based electrodes) to ~+0.8 V (*vs*. SCE) (utilising this printed 3DE) that can be associated to the reduced amount of edge plane sites available, which is in agreement with the results exhibited utilising the outer-sphere redox probe hexammineruthenium (III) chloride. Analytes that are renowned for their surface catalysed (C/O groups) were next analysed, namely, ascorbic acid and dopamine hydrochloride. ESI Fig. 6B and C exhibit cyclic voltammograms of the cycling of the 3DE within these solutions, where all of the peak-to-peak separations of the electrochemically active probes are presented in Table 3. In all cases this printed 3DE does not exhibit an increase within its electroactivity over traditionally based graphitic electrode materials reported within the literature^27^.

## 3D printed Lithium-ion Battery anode

**Figure 3:**
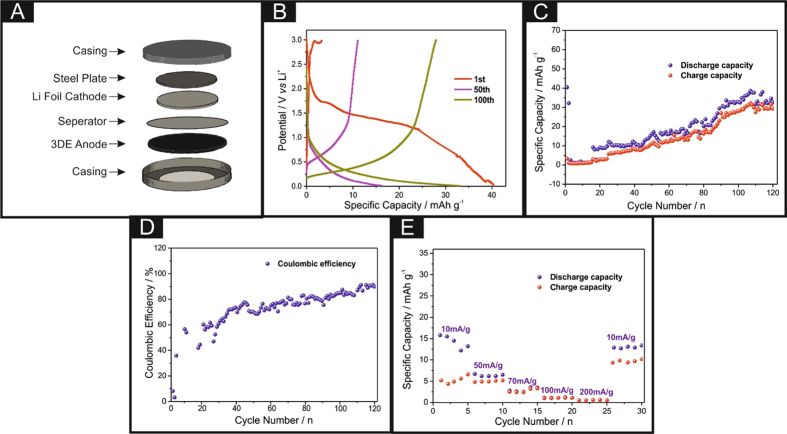
Schematic of the coin cell fabrication (**A**), charge–discharge profiles (**B**), cycling properties (**C**), coulombic efficiency (**D**) and rate capability of the 3D printed anode (**E**).

The CR2016-type coin cells were assembled in a glove box filled with dry argon atmosphere, which contained the lithium metal foil as a counter and reference electrode, and the polypropylene film (Celgard 2400) as a separator. 1 M solution of LiPF_6_ in 1:1 (v/v) mixture of ethylene carbonate (EC) and diethyl carbonate (DEC) was used as the electrolyte. Notably, graphene/PLA anodes were 3D printed with the same geometries as a CR-2016 coin cell (*i.e*. a diameter of 17.75 mm with a thickness of 1 mm) using a conventional RepRap 3D printer (as described within the Methods section). The utilisation of these 3D printed discs as a potential anode material within a lithium-ion coin cell were next considered. It is important to note that these freestanding anodes do not require a current collector and can simply slot into any coin cell configuration, as shown in Fig. 3A (and is comparatively shown in ESI Fig. 7). The cells were galvanostatically discharged and charged at different current densities between 3.0 and 0.01 V (*vs*. Li/Li^+^) using an Arbin battery cycler (BT2000, USA). Figure 3B depicts the voltage profiles for the 3DEs upon the 1^st^, 50^th^ and 100^th^ cycle, with relatively low discharge specific capacities of 40, 33 and 16 mAh g^−1^ respectively, however it is clear that there is a large irreversible capacity loss with the values corresponding to 5 mAh g^−1^. This sizeable deviation could be attributed to the high specific surface area of the 3DE and the formation of the solid electrolyte interface (SEI). Nevertheless, it is apparent that over time there is an improvement within the overall reversibility of the system. Additionally, upon the 1^st^ discharge/charge cycle the voltage presented is relatively large compared to that of other graphitic anode materials, with values of ~+2.25 V (*vs.* Li/Li^+^), which dissipates over time to the voltage region expected from a graphite based material, indicating that there could be metallic impurities residing upon the electrode surface. The cycling capability of the 3DE was next explored and is shown in Fig. 3C. It is clear that the rechargeable specific capacity over the first two cycles is relatively poor, which again can be attributed to a large amount of Li^+^ ions being unable to be extracted from the cavities within the 3DE. Upon cycle numbers 3–120, the rechargeable specific capacity improves, and a columbic efficiency of ~85% is achieved on the final scan, with an irreversible capacity reaching a maximum of 40 mAh g^−1^ (~8% of the overall weight accounts for the active graphene content in the entire 3D disc), if we consider the full weight of the printed 3DE the maximum specific capacity can only reach 3.69 mAh g^−1^. This is relatively low compared to other graphite-based anode materials, however it should be noted that the weight of the active material within this freestanding graphene/PLA anode is much larger than that of the typically deposited nanomaterials. For example, within the field an ‘ink’ composite is fabricated utilising a small amount of active material that is then mixed with binders and additives, which is then deposited in a uniform fashion upon a copper foil. The rate capabilities (Fig. 3E) of the 3DE were considered, with discharge capacities of 15.8, 6.2, 2.6, 1.1 and 0.6 mAh g^−1^ at current densities of 10, 50, 70, 100 and 200 mA g^−1^ respectively. Upon changing the current density back to 10 mAh g^−1^ the discharge capacity does recover to 13 mAh g^−1^.

Despite the low amount of conductive material within the composite, the overall performance of this 3D printed system within this proof-of-concept shows promising experimental data. The columbic efficiency and overall reversibility of the system could also indicate that there are some possible parasitic reactions occurring within the composite.

## 3D printed Solid-State Supercapacitor (3D-SC)

**Figure 4:**
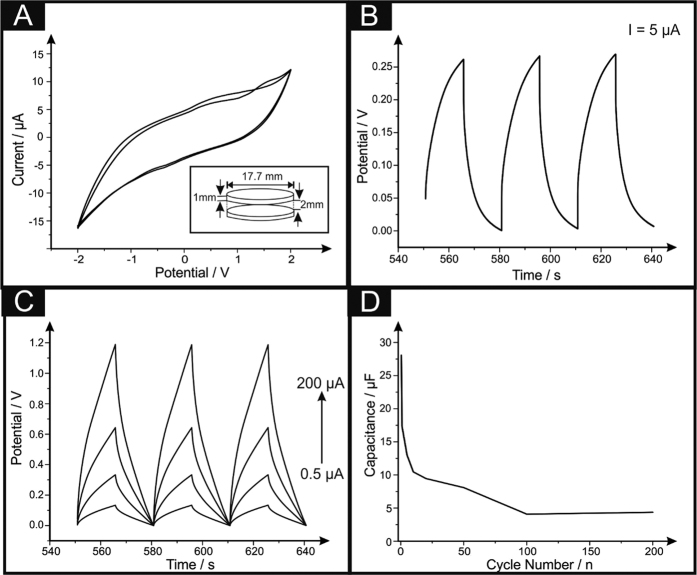
Cyclic voltammetry (**A**) of the 3D-SC consisting of a 2 mm layer of solid electrolyte of PVA and 1.0 M H_2_SO_4_. Corresponding charge/discharge curves with (**C**) and without (**B**) the Kampouris’ circuit in parallel are also presented. Scan Rate: 25 mV s^−1^. Inset to A is a schematic of the 3D-SC utilised throughout this study.

A 3D printed solid-state supercapacitor, 3D-SC, is developed to evaluate the potential of this 3D printable graphene filament utilising two 3D printed discs and sandwiching a solid electrolyte between the two, creating a fully freestanding supercapacitor. The solid electrolyte is prepared by mixing 6 g polyvinyl acetate (PVA) with 10 mL of 1.0 M H_2_SO_4_ (as mentioned in the Methods section), leaving a completely freestanding solid-state structure utilising 3DEs, depicted in the inset of Fig. 4A. Upon creation of the 3D-SCs, cyclic voltammetric analysis was carried out, with the PVA-H_2_SO_4_ acting as a solid-state electrolytic layer, over a range of −2.0 V to 2.0 V, at a scan rate of 25 mVs^−1^ and is depicted in Fig. 4A. The voltammogram provides a general analysis of the capacitive properties of the 3D-SC, in that the volume of the curve is indicative of the capacitance of the system. Herein, we visualise the curve intersect the zeroth potential line at ~±5.0 μA, indicating the charging current range available for the device. Next, the 3D-SCs capacitive performance was characterised *via* galvanostatic charge/discharge cycling over 200 cycles, and is described in terms of specific capacitance of the weight of the entire device, 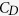, the weight of the working electrode, 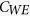, and the weight of the active material, graphene, in the working electrode, 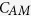. The characteristic saw-tooth charge-discharge behaviours are shown in Fig. 4B, for the 3D-SC with a charging current of 5.0 μA. The 3D-SCs exhibit consistent behaviour over the 200 cycles without any notable variation in the shape or range, consistently showing the same change in gradient over the potential range 0–0.25 V. Given the nature of the saw-tooth wave determining the gradient and hence capacitive properties of the material is complex. Therefore, a technique highlighted by Kampouris *et al*.^28^ is utilised to reduce any ambiguity of any values presented in Fig. 4C.

Attention was next turned towards obtaining the specific capacitance (*C*_*S*_) values of the 3D-SCs. Current literature utilises an array of methods in the calculation of *C*_*S*_, however the differences observed for each method are not reported. Thus, in this work a diverse range of methods were utilised to calculate the *C*_*S*_ values and Table 4 exhibits the differences observed. Method 1 is the typical analysis of entire device; method 2 evaluates the specific capacitance of the working electrode only; and method 3 indicates the specific capacitance associated with the active material only. In these equations, *C*_*Obs*_ is the observed capacitance (*F*) of the entire device. Furthermore, *m*_*Device*_ is the mass (*g*) of the entire device, both electrodes and the mass of the solid electrolyte layer. Also *m*_*WE*_ is the mass of the working electrode only and *m_Act_* is the mass of the active material in the working electrode *i.e*. graphene, assumed to be 8% of the total working electrode:

Method 1 for determining the capacitance of the device:


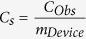


Method 2 for determining the capacitance of the working electrode:


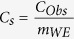


Method 3 for determining the capacitance of the active material:


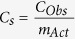


Table 4 exhibits specific capacity values for the whole device, working electrode and total active material (*i.e.* loading of 8% of total *wt*. confirmed by TGA), it is clear that the values, although not competitive with advanced nanomaterials, demonstrate the capabilities and potential for the fabrication of low cost, non-toxic 3D supercapacitative architectures.

## Application of the 3DE towards the Hydrogen Evolution Reaction (HER)

**Figure 5:**
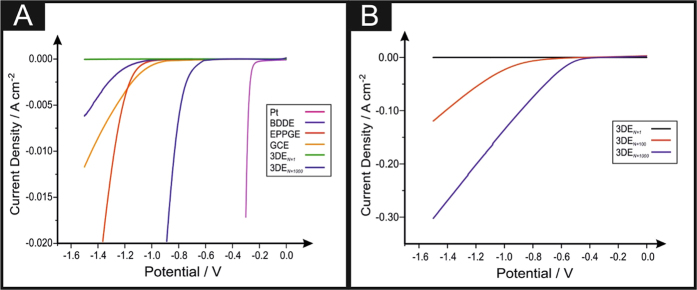
Comparative linear sweep voltammograms (LSV) (**A**) using 3DE compared to EPPGE, GCE, BDDE and platinum showing the onset of the HER. Stability studies of the 3DEs (**B**) using LSV for the initial, 10^th^, 100^th^ and 1000^th^ scans. Scan rate: 25 mV s^−1^ (*vs.* SCE). *Note: 3DE*_*N *=* 1*_ *is upon the initial scan and 3DE*_*N *=* 1000*_ *is upon the 1000*^*th*^ *scan*.

Research into 2D nanomaterials such as graphene have received enormous interest from a plethora of scientific disciplines into the exploration and exploitation of its unique properties; we next apply this 3D printed graphene structure towards the creation of hydrogen within an electrolyser^29^. The most common method of hydrogen production is the Hydrogen Evolution Reaction (HER) (2 H^+^ + 2e^−^ → H_2_). Platinum-based materials are commonly utilised for the HER, as this pure metal exhibits an extremely low binding energy of the H^+^ ions, which in turn allows for a low onset potential to occur^30^. As outlined in the introduction, one of the aims of this paper is it introduce a possible alternative to Pt. We therefore investigate the potential application of the 3DE fabricated herein as an electrode material towards the HER with the aim of revealing valuable insights into the 3DE’s electrocatalytic properties. Initially, it is essential to benchmark the electrochemical behaviour of the 3DE towards the HER and compare it to platinum and a range of bare/unmodified traditional carbon-based electrodes; namely, boron doped diamond (BDDE), edge plane pyrolytic graphite (EPPGE) and glassy carbon (GCE). Figure 5A shows linear sweep voltammetry (LSV) between 0 and −1.5 V for the initial scan of 3DE, the 1000^th^ scan of the 3DE, BDDE, EPPGE, GCE and platinum in 0.5 M H_2_SO_4_ as is common within the literature^31^. The HER activity observed for the 3DE is inferior, in regards to the observed HER onset −0.84 V (*vs*. SCE) to that of EPPGE and BDDE and at −0.78 V (*vs*. SCE) and −0.76 V (*vs*. SCE) whilst being superior to GCE at −1.05 V (*vs*. SCE). The current density achieved by the 3DE is negligible in comparison to all the other electrodes examined. Note that the HER onset is the potential at which the observed current initially begins to deviate from the background current. Whilst the 3DE proved to be relatively ineffectual as an electrocatalyst towards the HER it was essential to assess its electrochemical stability. This is a practical consideration for real world applications of 3DE as electrode materials where concerns over longevity and durability are paramount. It is evident that upon inspection of Fig. 5B that there is a decrease within the electronegative HER onsets and an increase in the current density corresponding to the increase in the number of LSV scans of which the 3DE undergoes. At the 100^th^ scan the HER onset is −0.70 V (*vs*. SCE) whilst the 1000^th^ scan the HER onset potential is −0.46 V (*vs*. SCE), which is the least electronegative all of the other carbon-based electrodes examined herein. The comparatively low overpotential for HER onset observed for the 1000^th^ scan of the 3DE is the closest observed to the desired value of platinum at −0.25 V (*vs*. SCE) thusly making it the most beneficial electrode towards the HER of all the carbon-based electrodes examined. It can therefore be theorised that the electrochemical reaction mechanism occurring has altered to account for this.

A common method to assess the HER reaction mechanism is *via* Tafel analysis. Tafel analysis was performed on the faradic regions of the LSV’s. Tafel slope values of ca. 167, 150 and 60 mV/dec^−1^ were determined for the 1^st^, 10^th^ and 1000^th^ 3DE scans. Interpretation of these values suggest that over the course of 1000 LSV scans the rate limiting step of the HER reaction mechanism on the 3DE changed from the “adsorption Volmer” step to most likely the “discharge Heyrosky step”, the observed change being indicative of the 3DE gaining an improved electrocatalytic prospectus. We postulate that when the 3DE is exposed to the acidic electrolyte, hydrolysation of the PLA chain occurs thus allowing a greater number of areas of reactive material to be revealed, which in this case we suspect is due to titanium-derived contaminants possibly created within the manufacturing stage of the graphene/PLA filament. Such hypothesis is compounded by the detection of titanium-based compounds revealed within both Raman and EDS analyses. Last, we have analysed the 3DE after its exposure to the acidic medium *via* XPS (presented in ESI Table 1), the resulting data indicates a lower proportion of oxygen bonding, which can be attributed to the hydrolysation of the PLA structure, in addition to this the formation of carbon-sulfur bonds has occurred due to exposure to the sulfuric acid.

## Conclusions

For the first time, proof-of-concept has been demonstrated utilising a printable 3D graphene-based PLA filament that has been 3D printed into useful electrochemical geometries. These systems are characterised both physicochemically and electrochemically, then are applied as *freestanding* lithium-ion anodes and solid-state graphene supercapacitors. Additionally, this 3D electrode (3DE) platform has been analysed towards its ability to create hydrogen *via* the hydrogen evolution reaction, in which these 3DEs exceed expectations and exhibit an extremely competitive onset potential compared to that of a platinum electrode. We believe that this platform (or similar) is the basis of next generation futuristic 3D printed energy architectures due to the following advantages:

1. 3D printing provides the fabrication of a *freestanding* electrochemical platform.
2. There is no need for a metallic current collector upon the Li-ion anode utilised, therefore offering a simplistic and low cost fabrication protocol for anode materials.
3. The thermoplastic supporting material allows the fabrication of an infinite amount of geometrical shapes and sizes, without the need for additional *ex-situ* post-curing/modification.
4. 3D printing of the filament can also improve the electrochemical behaviour with an increase of oxygenated species upon the surface of the 3D printed electrode platform.

In terms of this 3DE being used as a Li-ion anode and a solid-state supercapacitor the authors understand that the output is not highly competitive with current literature, however one must consider that in reality this anode/supercapacitor is comprised of *only* 8% graphene and 92% thermoplastic (PLA), and yet, still works as a battery anode/supercapacitor material! It should be noted that future work will examine a range of percentages and bespoke architectural structures.

## Methods

All chemicals used were obtained from Sigma-Aldrich at an analytical grade and were used without any further purification. All solutions were prepared with deionised water of resistivity not less than 18.2 MΩ cm. Voltammetric measurements were carried out using an Autolab PGSTAT100 (Metrohm, The Netherlands) potentiostat.

The 3D printed designs were fabricated using a RepRap printer with a direct drive extruder at a temperature of 210 °C, using a commercially procured filament, namely, Black Magic (Graphene/PLA) (Fig. 1A), with a calculated conductivity of 2.13 S/cm. The 3D printed designs were drawn *via* Solidworks, to create a circular disc electrode with a range of diameters with a thickness of 1.0 mm (Fig. 1B). The potentiostatic electrochemical experiments were carried out utilising a three-electrode setup with a printed 3D electrode (3DE) as the working electrode (with a diameter of 3 mm and a thickness of 1 mm) (Fig. 1C), a saturated calomel electrode (SCE) and platinum as the reference and counter electrodes respectively. Each 3DE for these experiments were printed with a connecting strip allowing simple connection to a crocodile clip.

CR2016-type coin cells were assembled inside a mBraun glovebox (H_2_O < 0.5 ppm, O_2_ < 0.5 ppm) using the metallic lithium counter/reference electrode, a polypropylene separator (Celgard 2400), an electrolyte of 1 M LiPF_6_ in ethylene carbonate and dimethyl carbonate (EC–DMC, 1:1) and a 3D printed graphene/PLA anode (with a diameter of 17.75 mm and a thickness of 1 mm). Charge–discharge measurements were carried out galvanostatically over a voltage range of 0.01–3.00 V using the Arbin battery test system (BT2000). A solid-state 3D printed supercapacitor (3D-SC) was developed utilising two 3D printed discs (as mentioned previously) and sandwiching a solid electrolyte between the two. The solid electrolyte is prepared by mixing 6 g polyvinyl acetate with 10 mL of 1.0 M H_2_SO_4_. The solution is heated to roughly 80 °C and mixed vigorously until a consistent gel is formed. A small sheet of polyester is wrapped around one disk providing a mould for the electrolyte gel, which is poured into the mould, with a 2 mm thick uniform coverage, the opposite electrode is forced into the mould before the solution solidifies. The resulting structure is left to cool for 24 hours, before the mould is removed, leaving a freestanding completely solid-state structure utilising 3DEs, the dimensions of which are illustrated in the inset of Fig. 4A. A small piece of copper wire is connected to each of the 3DEs using a conductive adhesive, to provide electrical connectors for long term testing. The 0.5 M H_2_SO_4_ solution used to explore the hydrogen evolution reaction (HER) was vigorously degassed prior to electrochemical measurements with high purity, oxygen free nitrogen.

Scanning electron microscope (SEM) images and surface element analysis were obtained with a JEOL JSM-5600LV model equipped with an energy-dispersive X-ray (EDX) microanalysis package. Raman Spectroscopy was performed using a Renishaw InVia spectrometer with a confocal microscope (×50 objective) spectrometer with an argon laser (514.3 nm excitation) at a very low laser power level (0.8 mW) to avoid any heating effects. Thermogravimetric analysis (TGA) was conducted utilising a PerkinElmer TGA 4000. The PLA samples were subject to a gradual temperature increase of 10 °C per minute, over a range between 25–800 °C, under a flow of nitrogen (40 ml/min). The X-ray photoelectron spectroscopy (XPS) data was acquired using a bespoke ultra-high vacuum system fitted with a Specs GmbH Focus 500 monochromated Al Kα X-ray source, Specs GmbH Phoibos 150 mm mean radius hemispherical analyser with 9-channeltron detection, and a Specs GmbH FG20 charge neutralising electron gun. Survey spectra were acquired over the binding energy range 1100–0 eV using a pass energy of 50 eV and high-resolution scans were made over the C 1 s and O 1 s lines using a pass energy of 20 eV. Under these conditions the full width at half maximum of the Ag 3d_5/2_ reference line is ∼0.7 eV. In each case, the analysis was an area-average over a region approximately 1.4 mm in diameter on the sample surface, using the 7 mm diameter aperture and lens magnification of ×5. The energy scale of the instrument is calibrated according to ISO 15472, and the intensity scale is calibrated using an in-house method traceable to the UK National Physical Laboratory. Data was quantified using Scofield cross sections corrected for the energy dependencies of the electron attenuation lengths and the instrument transmission. Data interpretation was carried out using CasaXPS software v2.3.16.

The values of the heterogeneous electron transfer rate constant, *k*^*0*^_*obs*_, were determined utilising the Nicholson method through the use of the following equation: *ψ* = *k*^*0*^_*obs*_[π*DnνF*/(*RT*)]^−1/2^ where *ψ* is the kinetic parameter, *D* is the diffusion coefficient, *n* is the number of electrons involved in the process, *F* is the Faraday constant, *R* is the universal gas constant and *T* is the temperature^32^. The kinetic parameter, *ψ*, is tabulated as a function of *ΔE*_*P*_ (peak-to-peak separation) at a set temperature (298 K) for a one-step, one electron process with a transfer coefficient, *α*, equal to 0.5. The function of *ψ (ΔE*_*P*_), which fits Nicholson’s data, for practical usage (rather than producing a working curve) is given by: *ψ* = (−0.6288 + 0.0021*X*)/(1 − 0.017*X*) where *X* = Δ*E*_P_ is used to determine *ψ* as a function of *ΔE*_P_ from the experimentally recorded voltammetry; from this, a plot of *ψ* against [π*DnνF*/(*RT*)]^−1/2^ allows the *k*^*0*^_*obs*_ to be readily determined^33^. The heterogeneous electron transfer rate constants were calculated assuming a diffusion coefficient of 9.10 × 10^−6 ^cm^2 ^s^−1^ for hexaammineruthenium (III) chloride^34^. To evaluate the effective electrochemical area, *A*_eff_, given that for a co-planar macro electrode in the electrochemically quasi-reversible case, the Randles–Ševčík equation (at 298 K): *I*_*p*_^*Quasi*^ = 2.65 × 10^5^*n*^3/2^*D*^1/2^*v*^1/2^[*C*]*A*_eff_, where the notation is the same as above and [*C*] is the concentration of electroactive substance, the geometrical surface of the area (*A*_*Geo*_) was calculated from the contact area of the graphene/PLA and printed 3DE immersed within the 1 mM hexaammineruthenium (III) chloride/0.1 M KCl solution. The real area percentage (*A*_*Real*_) was carried out utilising the following equation: *A*_*Real*_ (*%*)* *=* A*_*eff*_/*A*_*Geo*_ × 100.

## Additional Information

**How to cite this article**: Foster, C. W. *et al*. 3D Printed Graphene Based Energy Storage Devices. *Sci. Rep.* **7**, 42233; doi: 10.1038/srep42233 (2017).

**Publisher's note:** Springer Nature remains neutral with regard to jurisdictional claims in published maps and institutional affiliations.

## Supplementary Material

Supplementary Information

## Figures and Tables

**Table 1:** XPS analysis of industry standard PLA, graphene/PLA and the printed 3DE.

Element	Industry Standard PLA				Bulk Graphene/PLA				Printed 3DE			
	Elemental Atom %	Moiety			Elemental Atom %	Moiety			Elemental Atom %	Moiety		
		Assignment	BE (eV)	% of elemental signal		Assignment	BE (eV)	% of elemental signal		Assignment	BE (eV)	% of elemental signal
C 1 s	81.4	C—C	284.9	79.97	85.4	C—C	285.0	87.69	66.65	C—C	285.0	49.10
		C—O	287.3	6.63		C—O	287.6	3.47		C—O	287.6	26.01
		COO^−^	289.4	5.91		COO^−^	289.7	3.53		COO^−^	289.1	24.89
O 1 s	15.0	C—O	532.2	68.82	11.5	C—O	532.1	67.65	32.68	C—O	532.3	16.54
		C=O	533.7	31.18		C=O	533.6	31.73		C=O	533.7	15.62
N 1s	1.10	Organic N_2_	399.9	100	0.80	Organic N_2_	399.9	100	1.88	Organic N_2_	400.0	100

**Table 2:** A comparison of the electrochemical effective area *A*_eff,_ calculated *via* the quasi-reversible Randles–Ševčík equation (see Methods section) and the geometrical surface area *A*_Geo_, (calculated from the physical contact area immersed within 1 mM hexaammineruthenium (III) chloride/0.1 M KCl) of the graphene/PLA and printed 3DE. The real area percentage, *A*_Real_, is also presented for additional comparison (see Methods section).

Electrode	*A*_*Geo*_/cm^2^	*A*_eff_/cm^2^	*A*_Real_/%
Graphene/PLA	0.13	0.066	50.77
3DE	0.36	0.085	23.61

**Table 3:** The peak positions for the oxidation, *E*_*p*_^*Ox,*^ and reduction, *E*_*p*_^*Red*^, of an array of electroactive redox probes/analytes utilised in this study. Scan rate: 50 mV s^−1^.

Analytes	Voltammetric Peak Potentials/V (*vs.* SCE)		
	*E*_*p*_^*Ox*^	*E*_*p*_^*Red*^	*ΔE*_*p*_
Hexaammineruthenium (III) chloride/0.1 M KCl	−0.14	−0.31	0.16
Ammonium iron (II) sulfate/0.2 M HClO_4_	+1.32	−0.10	1.43
Nicotinamide adenine dinucleotide/pH 7.4 PBS	+0.82	N/A	N/A
Ascorbic acid/pH 7.4 PBS	+0.93	N/A	N/A
Sodium nitrite/pH 7.4 PBS	+1.39	N/A	N/A
Dopamine/pH 7.4 PBS	+0.71	N/A	N/A

**Table 4:** Comparison of capacitance and specific capacity for the 3D-SC over the range of applied currents (0.5–200 μA), calculated for the whole device, the working electrode (WE) and the active material within the printed 3DE (*i.e*. 8% graphene).

Current Applied/μA	Capacitance/μF	Specific Capacity/μF g^−1^		
		**Device**	**WE**	**Active Material**
0.5	28.07	17.17	63.11	485.47
1	17.36	10.62	39.03	300.28
5	13.00	7.95	29.23	224.87
10	10.46	6.40	23.52	180.93
20	9.45	5.78	21.25	163.51
50	8.08	4.94	18.18	139.84
100	4.08	2.49	9.180	70.61
200	4.36	2.67	9.817	75.51
