# Supplementary material for: 3D Printed Graphene Based Energy Storage Devices
Source: Sci Rep. 2017 Mar 3;7:42233. doi: 10.1038/srep42233 (PMC5361393; doi:10.1038/srep42233)

***Electronic Supporting Information***

**3D Printed Graphene Based Energy Storage Devices**

Christopher W. Foster ^a^, Michael P. Down ^a^, Yan Zhang ^b^, Xiaobo Ji ^b^,

Samuel J. Rowley-Neale ^a^, Graham C. Smith ^c^, Peter J. Kelly ^a^ and Craig E. Banks ^a^*

*^a^: Faculty of Science and Engineering, Manchester Metropolitan University, Chester Street, Manchester M15 GD, UK*

*^b^: College of Chemistry and Chemical Engineering, Central South University, Changsha 410083, China*

*^c^: Faculty of Science and Engineering, Department of Natural Sciences, University of Chester, Thornton Science Park, Pool Lane, Ince, Chester CH2 4NU, UK*

*To whom correspondence should be addressed.

Email: [c.banks@mmu.ac.uk](mailto:c.banks@mmu.ac.uk); Tel: ++(0)1612471196; Fax: ++(0)1612476831

Website:[www.craigbanksresearch.com](http://www.craigbanksresearch.com)**ESI Table 1:** XPS analysis of the printed 3DE after 1000 linear sweep cycles (*vs*. SCE) of the hydrogen evolution reaction (HER) within a solution of 0.5 M H_2_SO_4_. Scan rate: 25 mVs^-1^.

| **Element** | **Elemental Atom %** |
| --- | --- |
| C 1s | 62.3 |
| N 1s | 1.88 |
| O 1s | 30.72 |
| Si 2p | 0.74 |
| S 2p | 4.24 |

**ESI Figure 1**: Typical thermogravimetric curves for industry standard PLA (red line), graphene/PLA filaments (blue line) and the printed 3DE (black line).


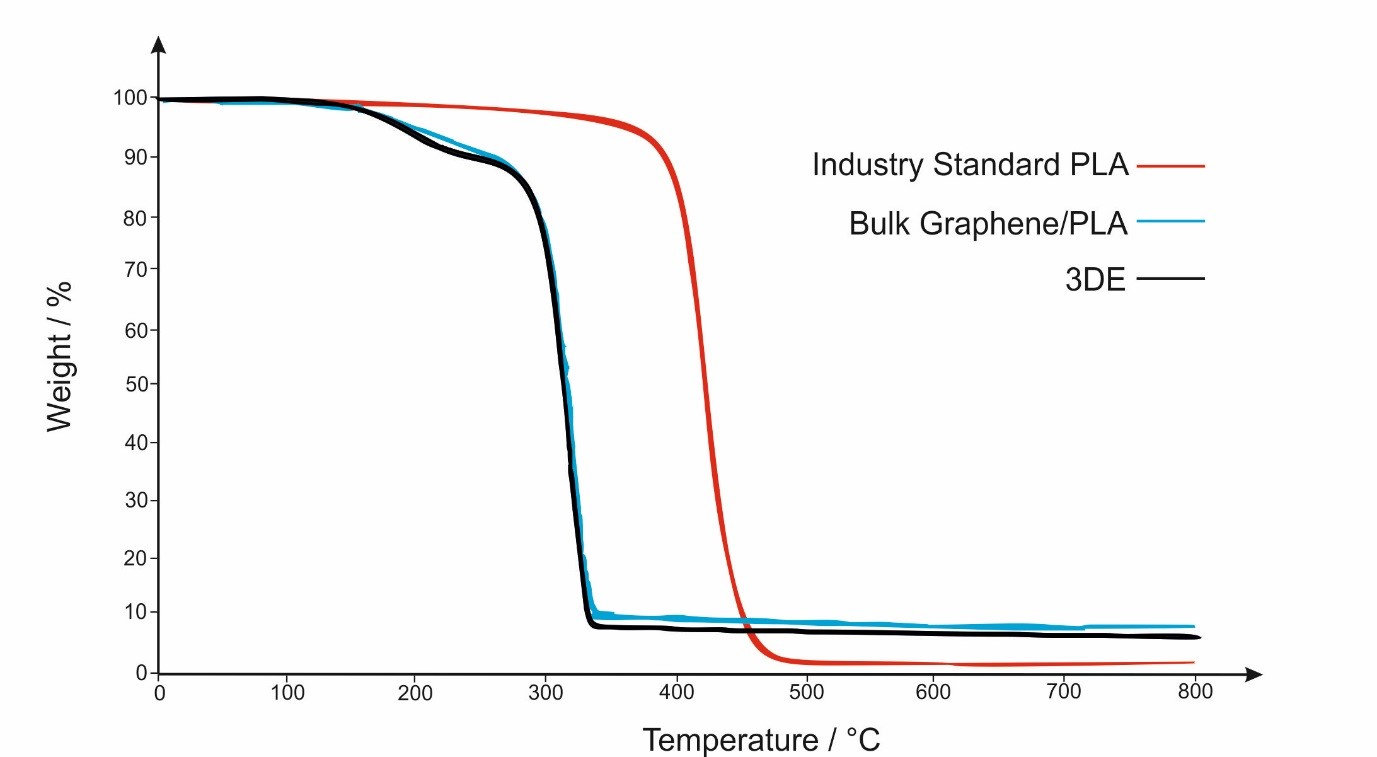


**ESI Figure 2:** SEM images of the graphene/PLA filament prior to any 3D printing.


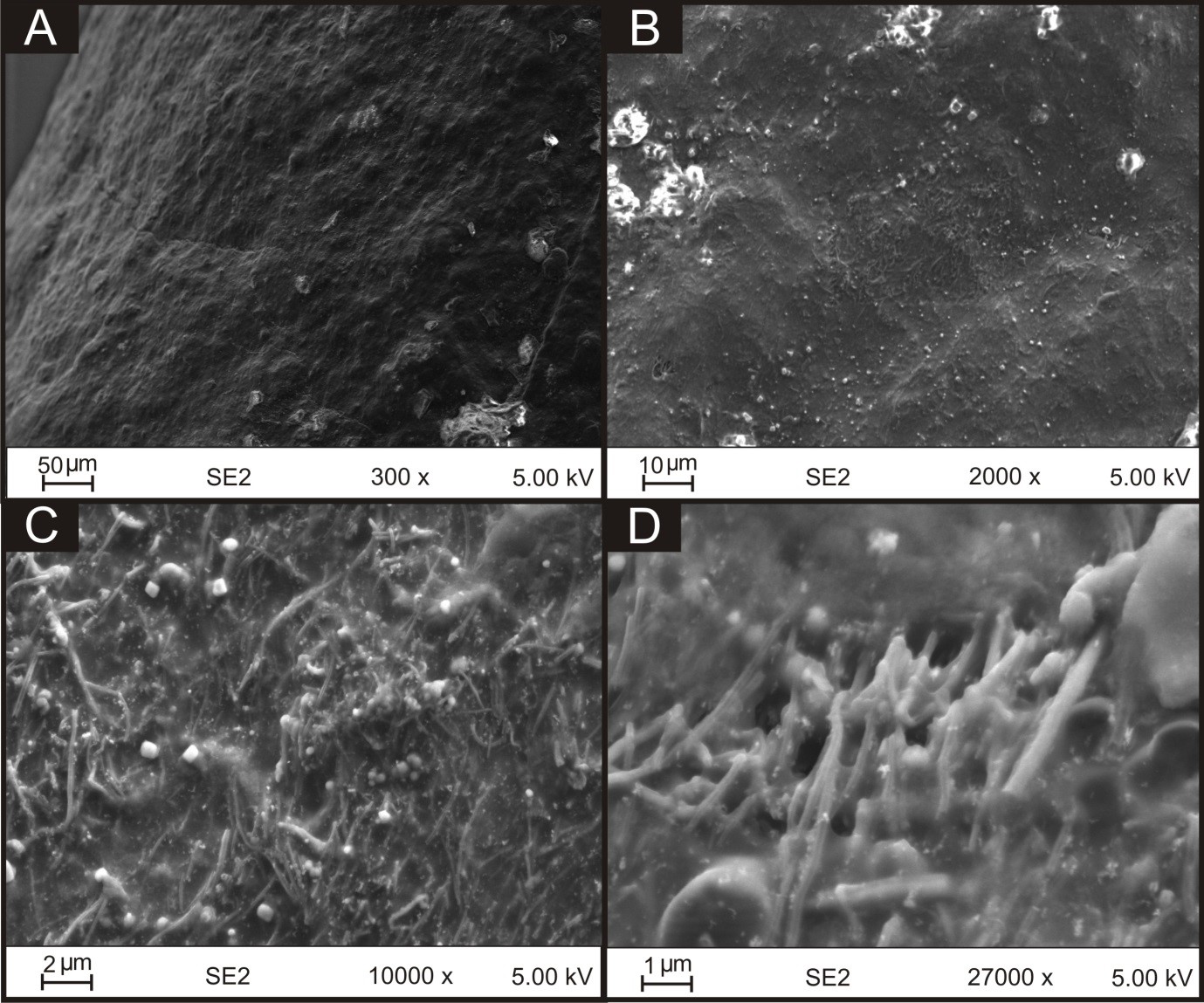


**ESI Figure 3:** Typical SEM images of the 3DE.


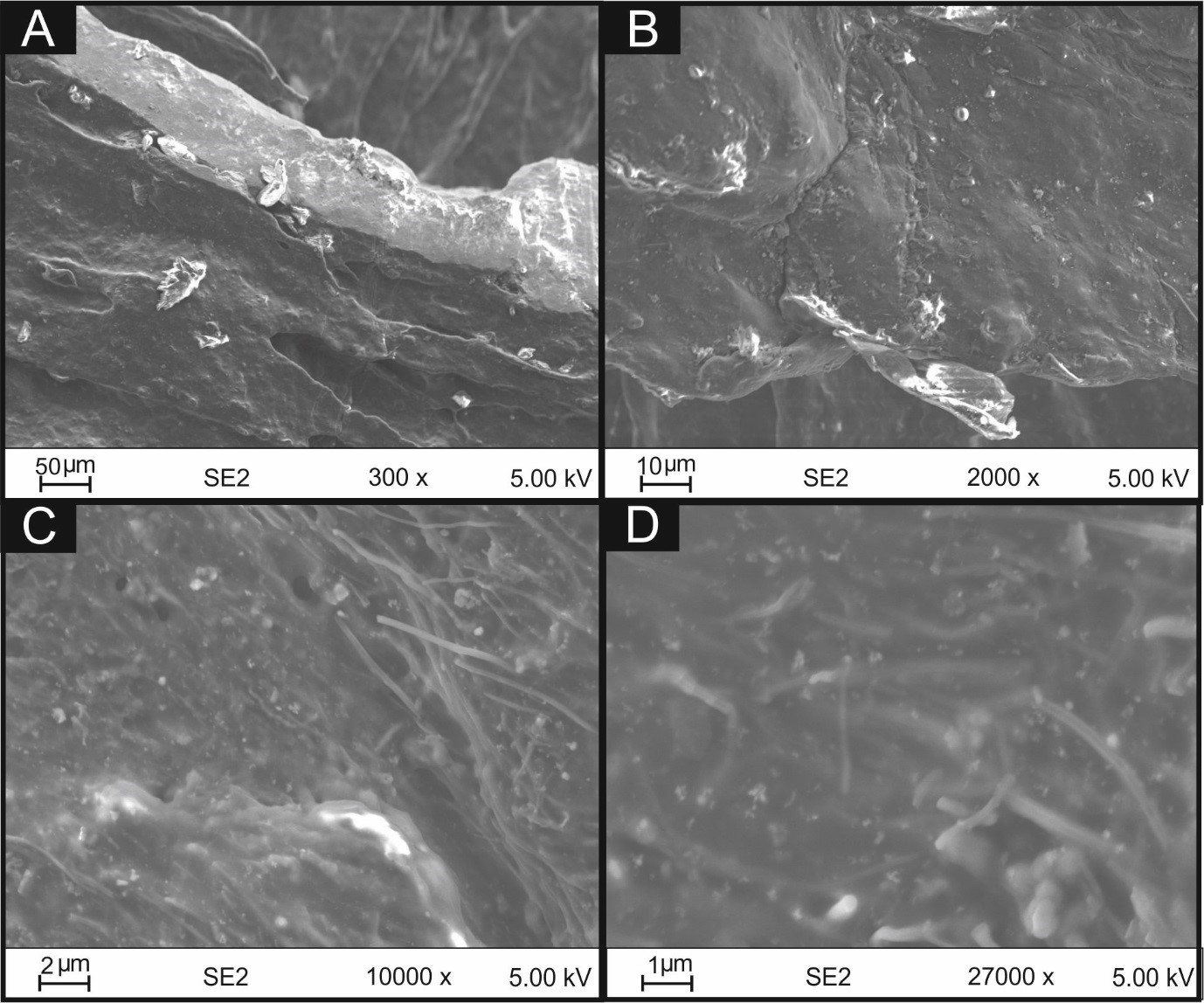


**ESI Figure 4:** EDS Spectra of the graphene/PLA filament of a cross section (purple line), external area (black line) and the printed 3DE (red line).


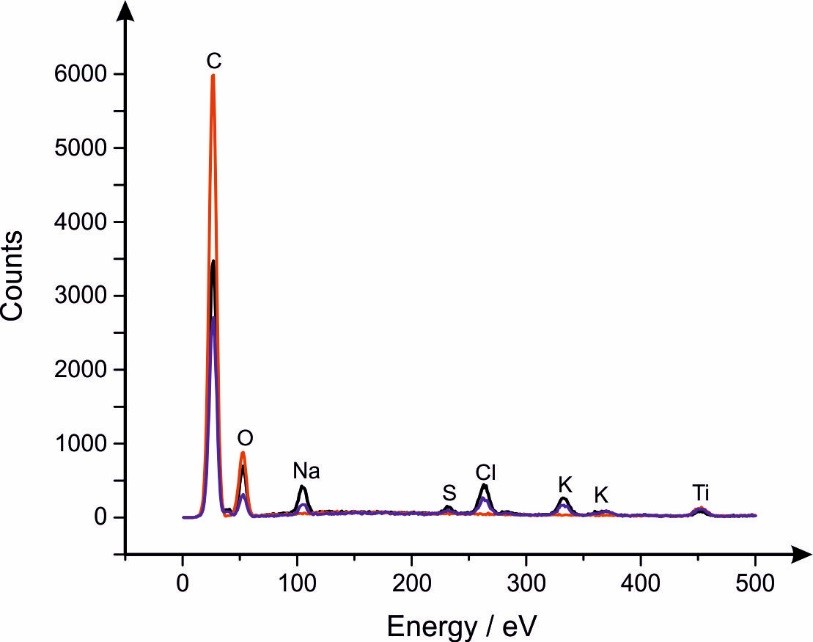


**ESI Figure 5**: Raman analysis of graphene/PLA (black line) and industry standard PLA (orange line) over the range of 1000 – 3200 cm^-1^ (A). Raman analysis of the printed 3DE over the range of 100 – 1000 cm^-1^ (B).


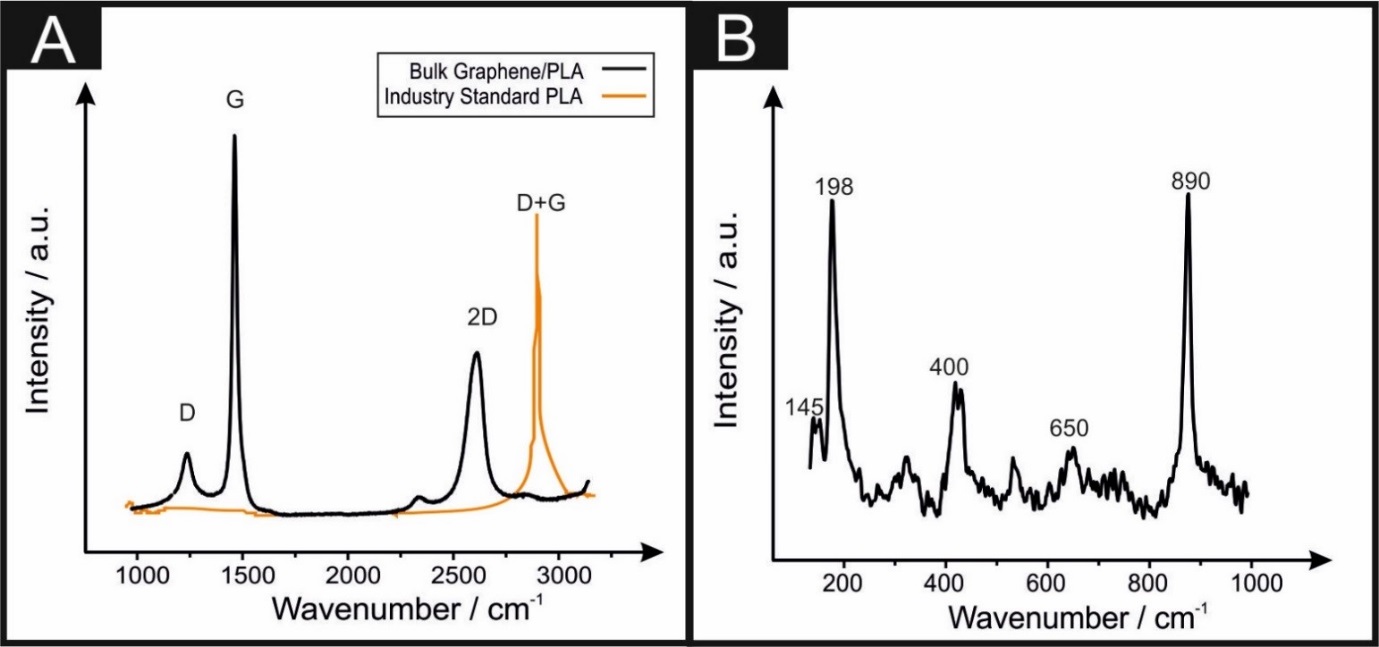


**ESI Figure 6**: Cyclic voltammetric responses of the printed 3DE (*vs*. SCE) explored towards the electrochemical oxidation of 1 mM NADH / pH 7.4 PBS (A), 1 mM ascorbic acid / pH 7.4 PBS (B), 1 mM dopamine / pH 7.4 PBS (C). First (solid line) and the second voltammetric scan (dashed line) are shown. Scan rate: 50 mV s^-1^.


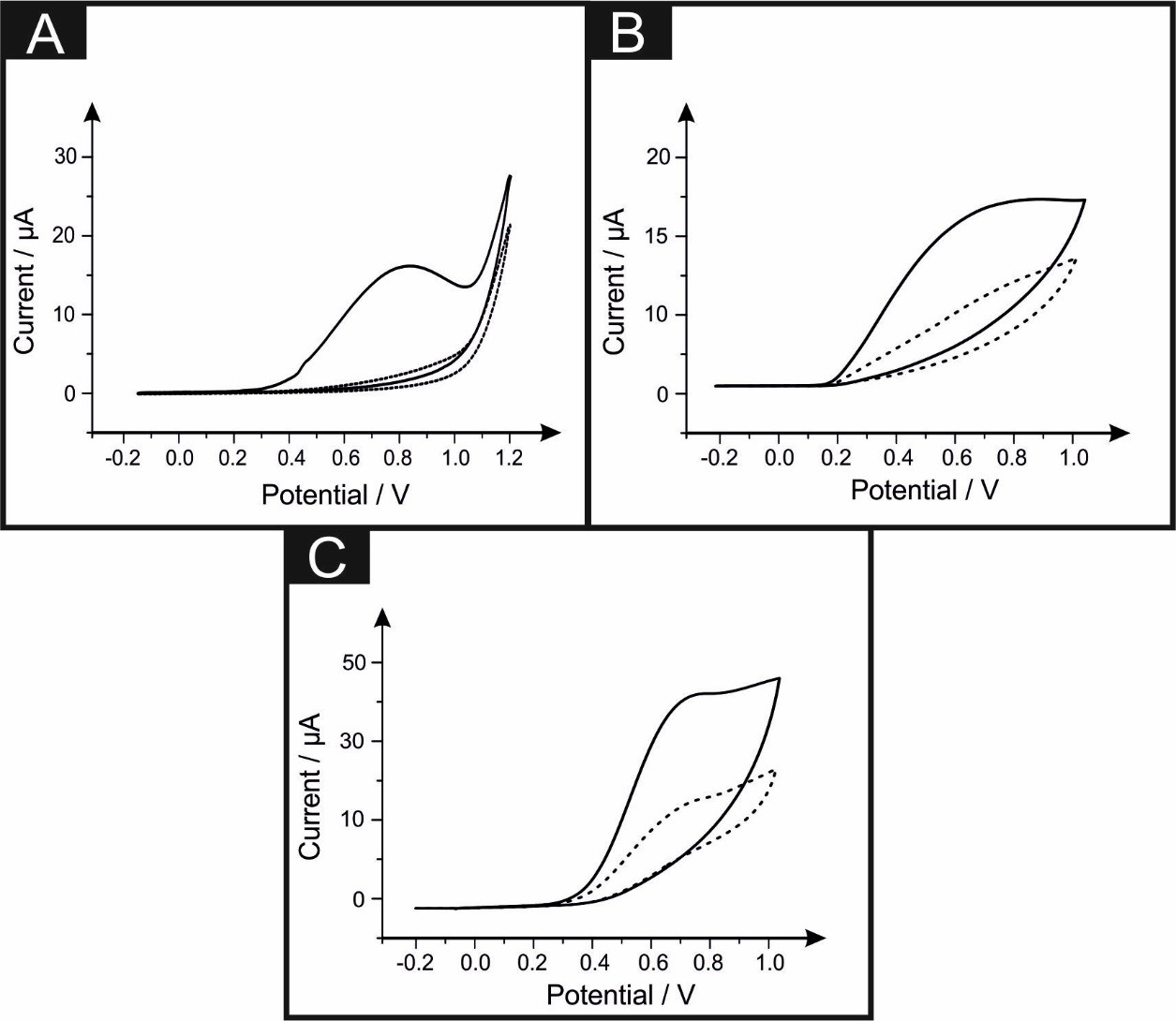


**ESI Figure 7:** Comparative schematics of the assembly of the coin cells used within this article (A) and the current literature (B), showing the advantages of the 3D printing concept/approach.


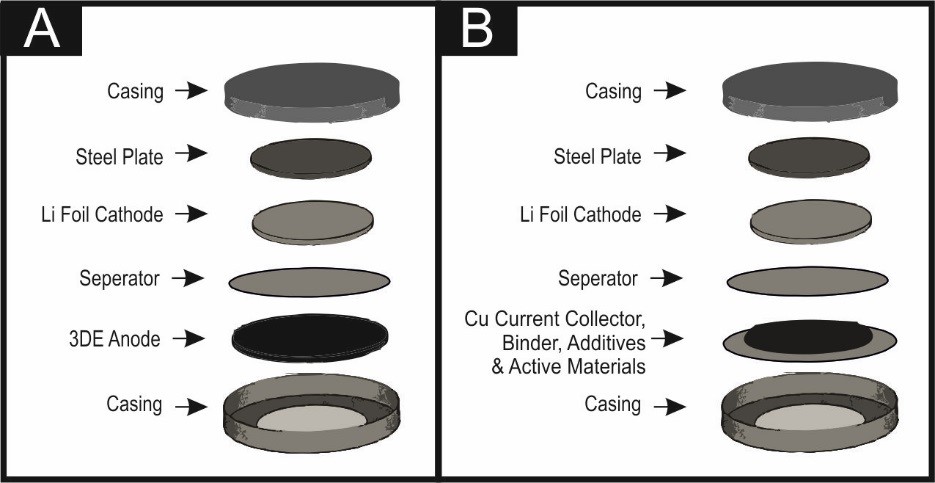

Supplement: Supplementary Information [file srep42233-s1.docx]
